# Clinical Value of a Routine Urine Culture Prior to Transrectal Prostate Biopsy

**DOI:** 10.1016/j.euros.2022.11.024

**Published:** 2022-12-26

**Authors:** Joakim Örtegren, Anna Wimmerstedt, Daniel Åberg, Håkan Janson, Henrik Kjölhede, Gunnar Kahlmeter, Ola Bratt

**Affiliations:** a Department of Urology, Institute of Clinical Sciences, Sahlgrenska Academy, University of Gothenburg, Gothenburg, Sweden; b Section of Urology, Department of Surgery, Region Kronoberg, Växjö, Sweden; c Department of Infectious Diseases, Region Värmland, Karlstad, Sweden; d Regional Office, Region Kronoberg, Växjö, Sweden; e Department of Clinical Microbiology, Region Kronoberg, Växjö, Sweden; f Department of Urology, Sahlgrenska University Hospital, Göteborg, Sweden

**Keywords:** Prostate, Biopsy, Infection, Urine culture, Antibiotic resistance

## Abstract

**Background:**

Infectious complications after a transrectal prostate biopsy may be severe. In Sweden, a routine culture prior to all prostate biopsies was introduced to enable targeted antimicrobial prophylaxis and reduce postbiopsy infections.

**Objective:**

To investigate whether a clinical routine with a urine culture prior to a prostate biopsy and targeted prophylactic antibiotic therapy reduces postbiopsy infections.

**Design, setting, and participants:**

In 2015, a site-specific antimicrobial stewardship programme with a urine culture prior to a prostate biopsy was initiated in Region Kronoberg. To evaluate this routine, we designed a population-based register study including all men who had an outpatient prostate biopsy in 2015–2019 and a control period including all men who had a biopsy in 2010–2014, when a urinary culture was obtained only on clinical suspicion.

**Outcome measurements and statistical analysis:**

The primary outcome was infectious complications within 10 d and the secondary outcome was a change in antibiotic prophylactic treatment. An infectious complication was defined as prescription of antibiotics for urinary tract infections or admission to hospital for urinary tract infections or sepsis after a biopsy.

**Results and limitations:**

The urine culture period included 2971 prostate biopsy procedures, of which 2684 (90%) were preceded by a urine culture*.* The control period included 2818 procedures, of which 135 (4.8%) were preceded by a urine culture. Infectious complications were slightly more common during the urine culture period (5.0%) than during the control period (4.3%, *p* = 0.17), as was inpatient care for infections (3.5% vs 2.2%, *p* = 0.002). The routine identified 5.4% men with asymptomatic bacteriuria. Despite targeted antibiotic treatment (1.5% received a nonfluoroquinolone treatment), the rate of infectious complications (6.3%) was similar to that in the control period.

**Conclusions:**

Prebiopsy urine culture did not lead to fewer postbiopsy infections. Other measures are needed to reduce infectious complications after a prostate biopsy.

**Patient summary:**

In this report, we evaluated a routine with urine culture prior to a transrectal prostate biopsy and found that it did not lead to fewer infectious complications.

## Take Home Message

We evaluated a clinical routine with urine culture prior to transrectal prostate biopsy in a comparative, population-based register study of almost 6000 procedures and found that it did not lead to fewer infectious complications.

## 1 Introduction

In Europe, >1 million prostate biopsies (PBs) are performed per year [1]. Infectious complications after a transrectal PB are an increasing problem. The proportion of patients having a febrile postbiopsy infection despite the use of prophylactic antibiotics varies across studies, from 0.7% to 7% [2], [3], [4], [5], [6]. The infection may cause severe sepsis and in rare cases even death [6]. The optimal prophylactic antibiotic regimen is not known. Until a few years ago, a single dose of an oral fluoroquinolone (FQ) was recommended and widely used [7], [8], [9], despite recommendations to avoid broad-spectrum and favour narrow-spectrum agents [10], [11], [12]. Owing to the increasing FQ resistance, recent awareness of potential serious side effects [13] and general overuse of broad-spectrum antibiotics, the European Association of Urology guidelines since 2021 instead recommend antibiotic prophylaxis based on a stool culture or rectal swab, augmented prophylaxis, or non-FQ antibiotics (fosfomycin, cephalosporin, or aminoglycoside) [14], [29], [30].

As asymptomatic bacteriuria is a risk factor for infectious complications after invasive procedures in the urinary tract, a urine culture and preprocedural, targeted antibiotics are recommended before many urological procedures [15]. A site-specific antimicrobial stewardship programme in Region Kronoberg, Sweden, included a routine culture prior to all PBs in the county from 2015 to enable targeted antimicrobial prophylaxis and reduce postbiopsy infections. However, the value of a such a routine is not known. According to a Swedish study, asymptomatic bacteriuria is a risk factor for a postbiopsy infection [16], but two previous small studies, one from France [17] and one from the USA [18], did not support this. Owing to these conflicting results, we designed a large, population-based study to evaluate the clinical benefit of a routine prebiopsy urine culture and targeted antimicrobial prophylaxis to reduce postbiopsy infections.

## 2 Patients and methods

### 2.1 Study design

Observational, population-based, register study with a historical control period.

### 2.2 Setting and study population

In 2015, as part of a regional review of surgical antibiotic prophylaxis usage and general antimicrobial stewardship, a routine urine culture prior to a scheduled outpatient PB was implemented at all urology units in Region Kronoberg, Sweden, with the aim of enabling targeted antimicrobial prophylaxis to reduce both ineffective use of FQ and infectious complications. Prior to 2015, a prebiopsy urine culture was required only on clinical suspicion of bacteriuria.

The standard prebiopsy antibiotic prophylaxis was, both before and after the implementation of the new routine, one per-oral dose of 750 mg ciprofloxacin or, in patients with FQ allergy, one dose of 800/160 mg sulfamethoxazole/trimethoprim. Patients with a positive urine culture had their biopsies rescheduled and received a course of antibiotic treatment, guided by the resistance pattern of the culture, if possible, with a non-FQ antibiotic. All positive urine cultures and mixed bacterial urine growth were assessed individually by the treating urologist. After the antibiotic treatment, a new urine culture was obtained. As a rule, a negative urine culture was required before proceeding to a biopsy, and at the time of a PB, standard prebiopsy prophylaxis as described earlier was given. The standard biopsy procedure during the entire study period was ten to 14 systematic cores after periprostatic injection of a local anaesthetic.

The present study included all men in Region Kronoberg (202 000 inhabitants) who had an outpatient transrectal PB at any of the county’s three urology units (Växjö Regional Hospital, Ljungby Hospital, and Gränsbygdskliniken) from January 1, 2010 to December 31, 2019. The study period was divided into a urine culture period from January 1, 2015 to December 31, 2019, when urine cultures were sampled routinely before PB, and a control period from January 1, 2010 to December 31, 2014, that is, before the routine of prebiopsy urine culture was initiated.

The electronic medical records (EMRs) in Region Kronoberg (Cambio Cosmic, Cambio Healthcare Systems, Linköping, Sweden) include health care documentation, as well as diagnosis and intervention codes for each outpatient visit and inpatient episode. The EMRs encompass both hospital units and all primary care units in the region. We included patients in the study who had a registered outpatient visit with the intervention code TKE00 or KEB00 (PB). Inpatient PB procedures were excluded because most patients selected for an inpatient PB have severe comorbidity and therefore received antibiotic treatment rather than prophylaxis. Patients whose urine was sampled from a catheter, urostomy, or nephropyelostomy were also excluded, as they often have resistant bacteria and should be considered for a pre-PB urine culture also in the absence of a clinical routine.

### 2.3 Data collection

Clinical data were obtained from the EMR system. Age at PB and any diagnosis of diabetes mellitus (E10/E11) or prostate cancer (C61) were registered as baseline characteristics. Medical records for all patients admitted to hospital 0–10 d after the PB were reviewed to ensure complete registration of complications and to define patients who received treatment for severe infections at an intensive care unit (ICU).

All urine and blood culture results from October 1, 2009 to January 31, 2020, including bacterial antibiotic susceptibility test results, were retrieved from the culture register at the Department of Clinical Microbiology.

The study was approved by the Swedish Ethical Review Authority (2020-02066).

### 2.4 Definitions

Any urine culture from 6 wk to 1 d before the date of the PB was defined as the routine urine culture (a urine culture sampled at the day of the PB could not guide treatment). Bacterial growth was quantified as ≥10^6^–<10^7^ colony-forming units (cfu) per litre (sparse growth), ≥10^7^–<10^8^ cfu/l (moderate growth), or ≥10^8^ cfu/l (abundant growth).

All urine cultures with growth of *Escherichia coli* were reported as positive regardless of bacterial quantity, whereas only abundant growth of other *Enterobacterales*, *Staphylococcus aureus*, *Pseudomonas* species, and enterococci was reported as a positive culture in asymptomatic individuals.

Infectious complications were defined as a prescription in primary or secondary care of an antibiotic that could be used for a urinary tract infection (UTI) 1–10 d after a PB (a prescription same day was regarded as prolonged prophylaxis), and/or a hospital admission for a UTI or a blood stream infection 0–10 d after a PB. We defined antibiotics that could be used for a UTI as Anatomic Therapeutic Chemical classification system group J01CA penicillin with extended spectrum, J01DB first-generation cephalosporins, J01DD third-generation cephalosporins, J01EA trimethoprim and derivates, J01EE combinations of sulphonamides and trimethoprim, J01MA FQs, and J01RA combination of antibacterial and J01XE nitrofuran derivates. Diagnoses considered signifying a postbiopsy infection were N39 UTI, N30 cystitis, T81 complication after intervention, and A41 sepsis. The definition of sepsis and septic shock changed during study period [19], and Sepsis-3 definition was used for registration. Serious infectious complications were defined as an infectious complication requiring in-hospital care.

### 2.5 Outcome measures

The primary outcome measure was the proportion of PB procedures followed by an infectious complication within 10 d. The secondary outcome measures were the proportion of urine cultures with FQ-resistant bacteria and change of prophylactic therapy before a PB because of the urine culture result. Any non-FQ antibiotic prescription after a positive urine culture was registered as a change in antibiotic prophylaxis.

### 2.6 Statistical analysis

Fisher’s exact test was used to compare proportions. A two-tailed *p* value of <0.05 was considered statistically significant. The analyses were done with R, version 4.1.2. The presentation of the study adheres to the STROBE checklist for observational cohort studies**.**

## 3 Results

**Fig. 1:**
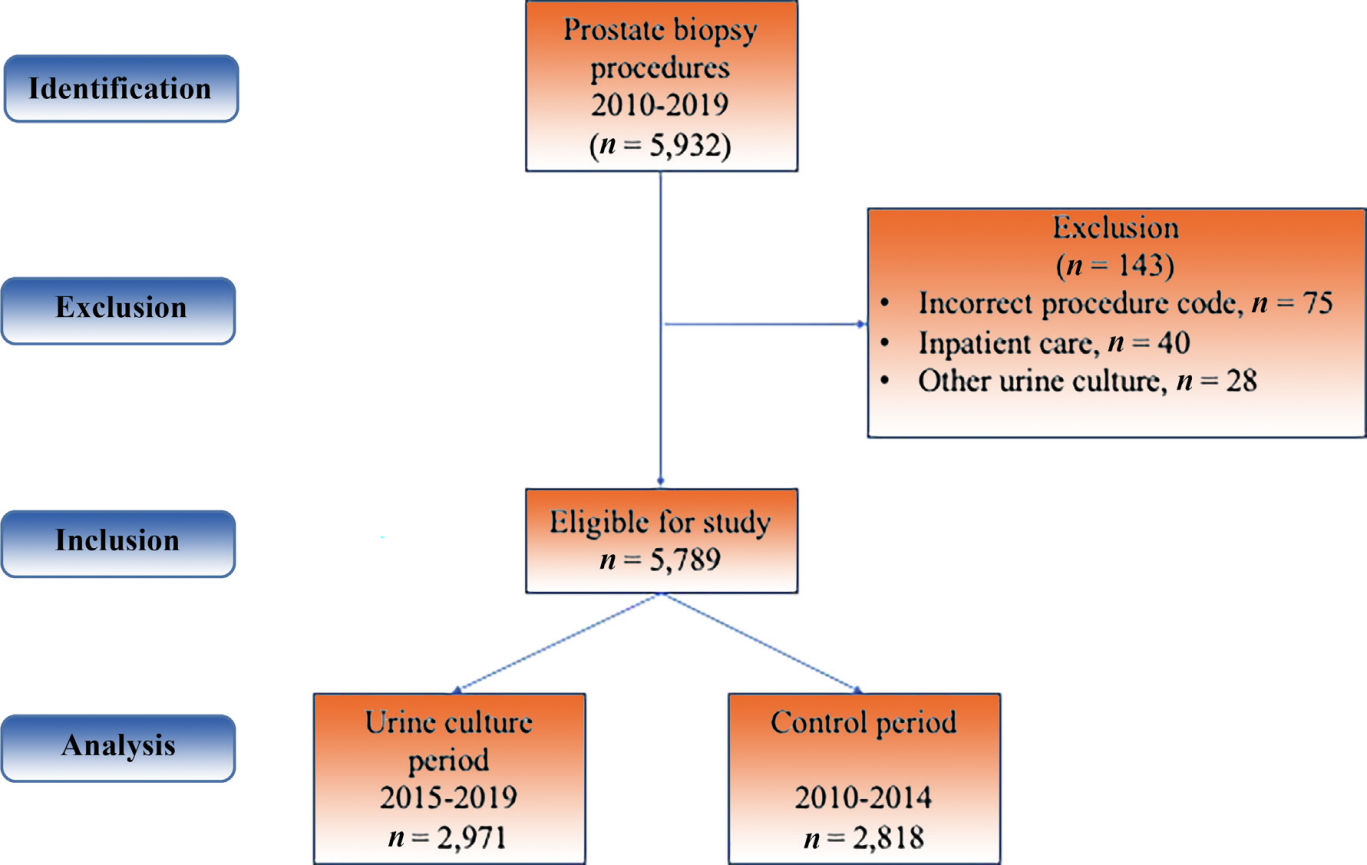
STROBE flow chart on study population.

A total of 5932 visits with a PB code were identified from January 1, 2010 to December 31, 2019. Of these, 143 (2.4%) were excluded: 75 because the procedure was incorrectly coded, 40 because the PB was done under inpatient care, and 28 because the urine sample was not voided, leaving 5789 PB procedures in 4041 patients for an analysis (Fig. 1). The urine culture period (2015–2019) included 2971 PB procedures, of which 2684 (90%) were preceded by a urine culture*.* The control period (2010–2014) included 2818 PB procedures, of which 135 (4.8%) were preceded by a urine culture. The age at PB and the proportions of men with diabetes were similar in both study periods, but the proportion of men who had a biopsy as part of active surveillance for a previously diagnosed prostate cancer was higher in the urine culture period (22%) than in the control period (15%). The patient characteristics at the time of PBs are shown separately for the two time periods in Table 1.

**Table 1:** Baseline characteristics at the time of prostate biopsy during the two study periods

	Prostate biopsy procedures	
	Urine culture period 2015–2019 *n* = 2971	Control period 2010–2014 *n* = 2818
Number of men	2292	2160
Age (yr), median (IQR)	67 (62–71)	68 (62–73)
Previous diagnosis of diabetes mellitus, *n* (%)	444 (16)	426 (14)
Previous diagnosis of prostate cancer, *n* (%)	664 (22)	431 (15)

IQR = interquartile range.

A post-PB infection was observed in a total of 150 patients (5.0%) undergoing PBs during the urine culture period and in 120 patients (4.3%) during the control period (*p* = 0.17). Inpatient care for a post-PB infection was more common during the urine culture period (3.5% vs 2.2%, *p* = 0.002). A positive blood culture was registered after 27 (0.9%) of the biopsy procedures during the urine culture period and after 15 (0.5%) procedures during the control period (*p* = 0.12). No patient met the sepsis criteria (SOFA-score ≥2) and none was treated at an ICU.

During the urine culture period, 160 (5.4%) of the pre-PB urine cultures were positive. All but one of these led to an antibiotic prescription: 113 (3.8% of all biopsy procedures) FQ and 46 (1.5%) non-FQ prescriptions. Of the 160 PBs preceded by a positive urine culture, ten (6.3%) were followed by an infectious complication. Five of the ten men received FQ and five non-FQ antibiotics. During the control period, 25 (0.9%) PBs were preceded by a positive urine culture, one of which was followed by an infectious complication. All 25 PBs with a positive urine culture resulted in an antibiotics prescription: 20 (0.7%) with FQ and five (0.2%) with non-FQ prescriptions (Table 2).

**Table 2:** Numbers of prostate biopsies preceded by urine culture, positive urine cultures, nonfluoroquinolone antibiotic treatments, and infectious complications after prostate biopsy in the two study periods

	Prostate biopsy procedures		*p* value
	Urine culture period 2015–2019 *n* = 2971	Control period 2010–2014 *n* = 2818	
PBs preceded by urine culture, *n* (%)	2684 (90)	135 (4.8)	
Positive urine cultures prior to PB, *n* (%)	160 (5.4)	25 (0.9)	
Infectious complication, *n* (%)	150 (5.0)	120 (4.3)	0.17
Admissions to hospital for UTI or sepsis 0–10 d after PB, *n* (%)	105 (3.5)	61 (2.2)	0.002
Prescriptions of antibiotic other than FQ prior to PB, *n* (%)	46 (1.5)	5 (0.2)	<0.001

FQ = fluoroquinolone; PB = prostate biopsy; UTI = urinary tract infection.

The proportion of FQ-resistant bacteria in the prebiopsy urinary cultures was 8.8% (14/160) in the urine culture period (*E. coli n* = 10, enterococci *n* = 4, and *S. aureus n* = 2) and 8.0% (2/25) in the control period. None of these men with FQ-resistant bacteriuria had an infectious complication.

## 4 Discussion

We evaluated a clinical routine with pre-PB urine cultures in a comparative, population-based register study of almost 6000 PB procedures and found that it did not lead to fewer infectious complications. On the contrary, infections leading to inpatient care were even more common after implementing routine pre-PB urine cultures.

Our results agree well with the results from the previous two small single-centre studies [17], [18], where positive prebiopsy urine cultures were left untreated without increased rates of infectious complications.

One possible reason for the higher post-PB infection rate in the urine culture period is that the proportion of patients on active surveillance was greater than that in the control period; previous research has shown that the risk of post-PB infections increases with the number of previous PBs [20]. The postbiopsy infection rate during the routine urine culture period in our study was similar to what was recently reported from a neighbouring county where they did not culture urine routinely before PB [21]. It is therefore unlikely that an increasing FQ resistance rate in the community would have caused an even higher post-PB infection rate in the absence of a routine pre-PB urine culture than was observed in our study. Indeed, the overall prevalence of FQ-resistant bacteria in positive urine cultures in Region Kronoberg was stable at around 10% in 2012–2019 [22].

The routine urine culture identified bacteriuria in 5.4% of the men before their planned PB, all of whom received targeted antibiotic treatment. One can assume that as many patients had undetected bacteriuria in the control period, and because post-PB infections were not more common in that period, it cannot be concluded that the targeted antibiotic treatment prevented any post-PB infection.

Of the 160 men with a positive urine culture prior to PB in the urine culture period, 14 had FQ resistance and were adequately treated without an infectious complication. Of the remaining 136 men with asymptomatic bacteriuria, ten (6.9%) suffered an infectious complication despite targeted treatment. It is thus possible that the routine with a pre-PB urine culture increased the risk of infections by leading to more complete FQ treatment courses that increased FQ resistance. The routine may also have harmed some men by delaying their cancer diagnosis.

Our study does not offer any evidence that isolated asymptomatic bacteriuria is a risk factor for post-PB infections. The results rather support the hypothesis that translocation of bacteria from faecal flora to the prostate and periprostatic tissues is the major cause. As the microbial reservoir of FQ-resistant bacteria is in the colon, future research in this area should focus on the clinical value of pre-PB faecal culture [23], rectal disinfection with povidone-iodine [24], and the transperineal biopsy route [25], [26].

The main strengths of our study are the population-based design, large study sample, high adherence to the pre-PB urine culture routine, and complete coverage of the EMRs for all parts of the health care services in the county. Limitations include the “before-and-after” comparison that, although reducing the risk of selection bias, made the results susceptible to confounding factors that caused an increased risk of post-PB infections for the patients who had a PB in the urine culture period. Another limitation is that we did not use a strict guideline for targeted antibiotic treatment. Additionally, we did not have any data on other risk factors such as FQ resistance in the faecal flora. Other limitations are the retrospective design and the short follow-up period of 10 d after a PB (some infectious complications may have occurred later). Moreover, the results of our study may be applicable only to populations with low FQ resistance. The prevalence of 10% in our region during 2012–2019 [22] is lower than that reported from other countries [27], [28]. Routine urine cultures before a PB may therefore be beneficial in populations with higher FQ resistance, despite the negative results from our study.

## 5 Conclusions

In conclusion, the routine with a urine culture prior to a PB did not reduce the postbiopsy infection rate. Future research should focus on other measures to reduce infectious complications after a PB.

  ***Author contributions*:** Joakim Örtegren had full access to all the data in the study and takes responsibility for the integrity of the data and the accuracy of the data analysis.

  *Study concept and design*: Örtegren, Bratt, Kjölhede, Wimmerstedt, Janson, Kahlmeter, Åberg.

*Acquisition of data*: Örtegren, Åberg.

*Analysis and interpretation of data*: Örtegren, Bratt, Kjölhede, Åberg.

*Drafting of the manuscript*: Örtegren, Bratt, Kjölhede, Wimmerstedt, Janson, Kahlmeter.

*Critical revision of the manuscript for important intellectual content*: Örtegren, Bratt, Kjölhede, Wimmerstedt, Janson, Kahlmeter.

*Statistical analysis*: Örtegren, Bratt, Kjölhede, Åberg.

*Obtaining funding*: Örtegren, Bratt, Kjölhede.

*Administrative, technical, or material support*: Örtegren.

*Supervision*: Örtegren, Bratt, Kjölhede, Kahlmeter, Janson.

*Other*: None.

  ***Financial disclosures:*** Joakim Örtegren certifies that all conflicts of interest, including specific financial interests and relationships and affiliations relevant to the subject matter or materials discussed in the manuscript (eg, employment/affiliation, grants or funding, consultancies, honoraria, stock ownership or options, expert testimony, royalties, or patents filed, received, or pending), are the following: None.

  ***Funding/Support and role of the sponsor*:** This research was supported by grants from Cancerstiftelsen Kronoberg and by grants from the Swedish Government under the agreement between the Swedish government and the county councils, the ALF-agreement (ALFGBG-873181).

## References

[1] Loeb S., Vellekoop A., Ahmed H.U. (2013). Systematic review of complications of prostate biopsy. Eur Urol.

[2] Bennett H.Y., Roberts M.J., Doi S.A.R., Gardiner R.A. (2016). The global burden of major infectious complications following prostate biopsy. Epidemiol Infect.

[3] Loeb S., Carter H.B., Berndt S.I., Ricker W., Schaeffer E.M. (2011). Complications after prostate biopsy: data from SEER-Medicare. J Urol.

[4] Danielsen L., Faizi G., Snitgaard S., Lund L., Frey A. (2019). Infections after transrectal ultrasonic guided prostate biopsies—a retrospective study. Scand J Urol Nephrol.

[5] Johansen T.E.B., Zahl P.-H., Baco E. (2020). Antibiotic resistance, hospitalizations, and mortality related to prostate biopsy: first report from the Norwegian Patient Registry. World J Urol.

[6] Alidjanov J.F., Cai T., Bartoletti R. (2021). The negative aftermath of prostate biopsy: prophylaxis, complications and antimicrobial stewardship: results of the global prevalence study of infections in urology 2010–2019. World J Urol.

[7] Lightner DJ, Wymer K, Sanchez J, Kavoussi L. AUA recommended antibiotic prophylaxis for urological procedures: table V. 2019 https://www.auanet.org/documents/Guidelines/PDF/Antimicrobial%20Prophylaxis%20Table%20V.pdf.

[8] Wagenlehner F.M.E., van Oostrum E., Tenke P. (2013). Infective complications after prostate biopsy: outcome of the Global Prevalence Study of Infections in Urology (GPIU) 2010 and 2011, a prospective multinational multicentre prostate biopsy study. Eur Urol.

[9] EAU. Guidelines on prostate cancer. 2019. https://uroweb.org/wp-content/uploads/EAU-EANM-ESUR-ESTRO-SIOG-Guidelines-on-Prostate-Cancer-2019-1.pdf.

[10] Tal R., Livne P.M., Lask D.M., Baniel J. (2003). Empirical management of urinary tract infections complicating transrectal ultrasound guided prostate biopsy. J Urol.

[11] Durham L.K., Ge M., Cuccia A.J., Quinn J.P. (2010). Modeling antibiotic resistance to project future rates: quinolone resistance in *Escherichia coli*. Eur J Clin Microbiol Infect Dis.

[12] Bell B.G., Schellevis F., Stobberingh E., Goossens H., Pringle M. (2014). A systematic review and meta-analysis of the effects of antibiotic consumption on antibiotic resistance. BMC Infect Dis.

[13] EMA. Disabling and potentially permanent side effects lead to suspension or restrictions of quinolone and fluoroquinolone antibiotics. 2019. https://www.ema.europa.eu/en/documents/press-release/disabling-potentially-permanent-side-effects-lead-suspension-restrictions-quinolone-fluoroquinolone_en.pdf.

[14] EAU guidelines. Presented at the EAU Annual Congress Amsterdam; 2022.

[15] Bonkat G BR, Bruyère F, Cai T, Geerlings SE, Köves F, Schubert S, Wagenlehner F. The European Association of Urology: guidelines on urological infections. 2021. http://uroweb.org/guidelines/compilations-of-all-guidelines/. 10.1016/j.eururo.2024.03.035 38714379

[16] Lindstedt S., Lindström U., Ljunggren E., Wullt B., Grabe M. (2006). Single-dose antibiotic prophylaxis in core prostate biopsy: impact of timing and identification of risk factors. Eur Urol.

[17] Bruyère F., D'Arcier B.F., Boutin J.M., Haillot O. (2010). Is urine culture routinely necessary before prostate biopsy?. Prostate Cancer Prostatic Dis.

[18] Qi D.Z., Lehman K., Dewan K., Kirimanjeswara G., Raman J.D. (2018). Preoperative urine culture is unnecessary in asymptomatic men prior to prostate needle biopsy. Int Urol Nephrol.

[19] Singer M., Deutschman C.S., Seymour C.W. (2016). The Third International Consensus definitions for sepsis and septic shock (Sepsis-3). JAMA.

[20] Bokhorst L.P., Lepistö I., Kakehi Y. (2016). Complications after prostate biopsies in men on active surveillance and its effects on receiving further biopsies in the Prostate cancer Research International: Active Surveillance (PRIAS) study. BJU Int.

[21] Forsvall A., Jönsson H., Wagenius M., Bratt O., Linder A. (2021). Rate and characteristics of infection after transrectal prostate biopsy: a retrospective observational study. Scand J Urol Nephrol.

[22] Department of Clinical Microbiology, Region Kronoberg. Resistensuteckling Kronoberg och Blekinge län (Swedish). 2020. https://www.mikrobiologi.org/resistensutveckling.

[23] Scott S., Harris P.N., Williamson D.A., Liss M.A., Doi S.A.R., Roberts M.J. (2018). The effectiveness of targeted relative to empiric prophylaxis on infectious complications after transrectal ultrasound-guided prostate biopsy: a meta-analysis. World J Urol.

[24] Pu C., Bai Y., Yuan H. (2014). Reducing the risk of infection for transrectal prostate biopsy with povidone–iodine: a systematic review and meta-analysis. Int Urol Nephrol.

[25] Pradere B., Veeratterapillay R., Dimitropoulos K. (2021). Nonantibiotic strategies for the prevention of infectious complications following prostate biopsy: a systematic review and meta-analysis. J Urol.

[26] Pilatz A., Veeratterapillay R., Dimitropoulos K. (2021). European Association of Urology position paper on the prevention of infectious complications following prostate biopsy. Eur Urol.

[27] Dalhoff A. (2012). Global fluoroquinolone resistance epidemiology and implications for clinical use. Interdiscip Perspect Infect Dis.

[28] Colpan A., Johnston B., Porter S. (2013). *Escherichia coli* sequence type 131 (ST131) subclone H30 as an emergent multidrug-resistant pathogen among US veterans. Clin Infect Dis.

[29] EAU. Guidelines on prostate cancer. 2021. https://uroweb.org/guidelines/prostate-cancer/chapter/diagnostic-evaluation.

[30] Bonkat G., Pilatz A., Wagenlehner F. (2019). Time to adapt our practice? The European Commission has restricted the use of fluoroquinolones since March 2019. Eur Urol.

